# Nomogram for Predicting Occult Locally Advanced Esophageal Squamous Cell Carcinoma Before Surgery

**DOI:** 10.3389/fsurg.2022.917070

**Published:** 2022-06-14

**Authors:** Zhixin Huang, Zhinuan Hong, Ling Chen, Mingqiang Kang

**Affiliations:** ^1^ Department of Thoracic Surgery, Fujian Medical University Union Hospital, Fuzhou, China; ^2^ Key Laboratory of Cardio-Thoracic Surgery (Fujian Medical University), Fujian Province University, Fuzhou, China; ^3^ Key Laboratory of Ministry of Education for Gastrointestinal Cancer, Fujian Medical University, Fuzhou, China; ^4^ Fujian Key Laboratory of Tumor Microbiology, Fujian Medical University, Fuzhou, China; ^5^ Department of Cardiac Surgery, Fujian Medical University Union Hospital, China

**Keywords:** occult lymph node metastasis, esophageal squamous cell carcinoma, predictor factors, nomogram, neoadjuvant therapy

## Abstract

**Introduction:**

The limitations of preoperative examination result in locally advanced esophageal squamous cell carcinoma (ESCC) often going undetected preoperatively. This study aimed to develop a clinical tool for identifying patients at high risk for occult locally advanced ESCC; the tool can be supplemented with preoperative examination to improve the reliability of preoperative staging.

**Materials and Methods:**

Data of 598 patients who underwent radical resection of ESCC from 2010 to 2017 were analyzed. Logistic multivariate analysis was used to develop a nomogram. The training cohort included patients who underwent surgery during an earlier period (*n* = 426), and the validation cohort included those who underwent surgery thereafter (*n* = 172), to confirm the model’s performance. Nomogram discrimination and calibration were evaluated using Harrell's concordance index (C-index) and calibration plots, respectively.

**Results:**

Logistic multivariate analysis suggested that higher preoperative carcinoembryonic antigen levels (>2.43, odds ratio [OR]: 2.093; 95% confidence interval [CI], 1.233–2.554; *P *= 0.006), presence of preoperative symptoms (OR: 2.737; 95% CI, 1.194–6.277; *P *= 0.017), presence of lymph node enlargement (OR: 2.100; 95% CI, 1.243–3.550; *P *= 0.006), and advanced gross aspect (OR: 13.103; 95% CI, 7.689–23.330; *P *< 0.001) were independent predictors of occult locally advanced ESCC. Based on these predictive factors, a nomogram was developed. The C-indices of the training and validation cohorts were 0.827 and 0.897, respectively, indicating that the model had a good predictive performance. To evaluate the accuracy of the model, we divided patients into high-risk and low-risk groups according to their nomogram scores, and a comparison was made with histopathological data.

**Conclusion:**

The nomogram achieved a good preoperative prediction of occult locally advanced ESCC; it can be used to make rational therapeutic choices.

## Introduction

Globally, esophageal cancers (EC) are among the malignant tumors with the highest morbidity and mortality (1), and esophageal squamous cell carcinoma (ESCC) accounts for the majority of EC (2). At present, surgery is still the most effective treatment for ESCC. However, studies have shown that patients with tumors staged above T3N0 or any TN1 (locally advanced esophageal cancer) were considered suitable for neoadjuvant therapy, and this has been recognized in most guidelines (3, 4). Performing surgery blindly may not yield many benefits, and may lead to unresectable tumors during surgery, thus wasting medical resources. Therefore, the accurate judgment of the clinical stage of patients with ESCC before surgery is a practical issue.

Currently, the mainstream practice in predicting the clinical stage of tumors is the use of computed tomography (CT), endoscopic ultrasound (EUS), and positron emission tomography-computed tomography (PET-CT). EUS is considered the current standard for preoperative T-staging of EC (5). However, EUS is still in its infancy in many parts of the world. Especially for patients in developing regions, EUS is an expensive and difficult examination method. Generally, only 10% of EUS procedures carried out are for upper gastrointestinal indications. Yoshinaga et al. reviewed the publications of Asian countries, except for Japan, China, South Korea, and Taiwan, and found that only 64 hospitals had EUS (6). PET-CT is also not popular in the diagnosis and treatment of esophageal cancer. In the final analysis, the cost of PET-CT was found to be difficult to bear for patients in developing countries. In China's guidelines for the diagnosis and treatment of esophageal cancer, PET-CT examination is of low priority. Moreover, several studies have shown that the incidence of occult lymph node metastasis is between 16% and 39% (7, 8), which means that several patients may choose inappropriate treatments. This questions the reliability of preoperative assessment for ascertaining an “early” decision. The ability to accurately identify occult locally advanced ESCC before surgery is worthy of consideration by clinicians. Unfortunately, there are only a few studies on this. Marco et al. believe that preoperative carcinoembryonic antigen (CEA) and CA19-9 levels are of value in identifying occult locally advanced EC (9). However, they did not include other factors in their analyses and did not establish a systematic predictive model to predict the risk of occult locally advanced EC.

A nomogram is a statistical model used in various medical fields for analysis (10). It is based on logistic multivariate analysis or multivariate Cox proportional hazards regression, and thus, integrates and quantifies multiple predictors. Compared with other prediction methods, nomograms can more intuitively and concisely analyze and quantify multiple clinical and pathological indicators for individualized assessment.

This study aimed to develop a predictive model for the risk of occult locally advanced ESCC in patients with negative preoperative lymph node examinations to assist in clinical diagnosis and treatment.

## Patients and Methods

### Study Design

**Figure 1:**
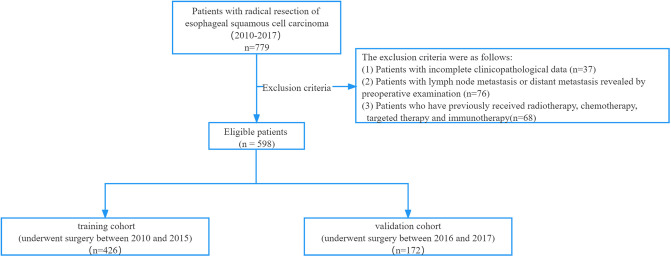
Study flow chart.

We retrospectively analyzed the clinical data of 779 patients treated at Fujian Medical University Union Hospital from 2010 to 2017. The inclusion criteria were as follows: (1) patients who underwent radical resection of EC and (2) patients with a postoperative pathological type of squamous cell carcinoma. The selected patients all had no distant metastasis clinically, as assessed by preoperative CT and other examinations (cT1-3N1-2M0). The exclusion criteria were as follows: patients with incomplete clinicopathological data (*n* = 37), patients with lymph node metastasis or distant metastasis revealed by preoperative examination (*n* = 76), or patients who previously received radiotherapy, chemotherapy, targeted therapy, and immunotherapy (*n* = 68). A total of 598 patients were included in this study. Eligible patients who underwent surgery between 2010 and 2015 were included in the training cohort for the development of the nomogram, and those who underwent surgery between 2016 and 2017 were entered into the validation cohort (Figure 1).

The requirement for obtaining patient informed consent was waived because the patient data remained anonymized. All procedures followed were in accordance with the ethical standards of the responsible committee on human experimentation (institutional and national) and with the Helsinki declaration of 1964 and its later versions.

### Preoperative Examination

Preoperative examinations, including high-resolution CT of the thorax and upper abdomen, abdominal color ultrasound, upper gastrointestinal tract radiography, color doppler ultrasound of the supraclavicular lymph nodes, and CEA test, were conducted in all patients. For carcinomas of the upper intrathoracic esophagus, neck CT was added. Digestive endoscopy was used to identify the pathology, pathological types, and gross aspect of tumors. The indications for surgery were evaluated by experienced thoracic surgeons.

### Relevant Definitions

The postoperative tumor staging was determined using the International Union Against Cancer Tumor–Node–Metastasis (TNM) classification and based on postoperative tumor staging as the outcome indicator and gold standard. Most guidelines recommend that patients with T1/T2 and lymph node metastasis or T3/T4 should receive neoadjuvant therapy first, and we classified these stages as locally advanced (11, 12) (Table 1). The gross aspect of the tumor was classified using the Paris classification (13) (Table 2). In China, superficial EC uses the Paris classification, wherein the medullary, mushroom, ulcer, and restricted intracavitary types correspond to the advanced type. Due to differences in the writing habits of endoscopy doctors, the report, which did not describe the gross aspect, imputed the missing values based on postoperative specimens. The criteria for judging lymph node enlargement were as follows: (1) Neck: the largest transverse diameter of the lymph node is ≥15 mm in the I–IV area, and the largest transverse diameter of the lymph node is ≥10 mm in the mediastinum (area VII); (2) Mediastinum: the lymph nodes in the five groups and the posterior area of the phrenic foot were assessed using a diagnostic threshold of 6 mm, and the other groups were assessed using a diagnostic threshold of 10 mm; (3) Abdominal cavity: a diameter ≥10 mm was used. Metastatic lymph nodes were excluded by experienced doctors in the Ultrasound Department of Fujian Union Medical College Hospital. The location of an esophageal tumor was assessed comprehensively based on the results of the preoperative examination, and the tumor was bounded by the upper pole. The preoperative symptoms mainly included dysphagia, as well as choking, foreign body, stagnation, and pain sensations behind the breastbone.

**Table 1:** Early-advanced ESCC classification.

Stage	pT	pN	Classification
Stage0	tis	N0	Early
Stage IA	T1a	N0	
	T1a	N0	
Stage IB	T1a	N0	
	T1b	N0	
	T1b	N0	
	T2	N0	
Stage IIA	T2	N0	
	T2	N0	
	T3	N0	Advanced (event point)
	T3	N0	
Stage IIB	T3	N0	
	T3	N0	
	T3	N0	
	T1	N1	
Stage IIIA	T1	N2	
	T2	N1	
Stage IIIB	T2	N2	
	T3	N1-2	
	T4a	N0-1	
Stage IVA	T4a	N2	
	T4b	N0-2	
	any T	N3	

**Table 2:** The goss aspect classification of digestive-tract cancer.

Type	Describe	Classification
Superficial type 0	Superficial protruding or non-protruding lesions	early gross aspect
Advanced type 1	Protruding carcinoma, attached on a wide base	advanced gross aspect
Advanced type 2	Ulcerated carcinoma with sharp and raised margins	
Advanced type 3	Ulcerated carcinoma without definite limits	
Advanced type 4	Nonulcerated, diffusely infiltrating carcinoma	
Advanced type 5	Unclassifiable advanced carcinoma	

### Statistical Analysis

All statistical analyses were performed using SPSS version 25 and R version 4.0.5. Continuous variables are expressed as mean ± standard deviation (SD) and compared using an unpaired two-tailed t-test or Mann–Whitney U test. Categorical variables were compared using the *χ^2^* test or Fisher’s exact test. Logistic regression analysis was used to determine the independent predictive factors for occult locally advanced ESCC. Variables with *P *< 0.1 in the univariable analysis were included in the multivariable model, which was used to estimate the odds ratio (OR) and the corresponding 95% confidential interval (CI) for every potential predictive variable in a forward stepwise manner. Statistical significance was set at *P *< 0.05. Based on the results of the multivariate logistic regression analysis of the training cohort, the nomogram was created using the RMS package of R 4.0.5 (http://www.r-project.org/).

Nomogram accuracy was determined using discrimination and calibration evaluations. Discrimination refers to the model's ability to distinguish patients with different results and uses the C-index and receiver operating characteristic (ROC) curves as measurement tools and calibration with 1,000 bootstrap samples to decrease overfit bias (14). The Brier score and calibration curve were used to evaluate the calibration ability of the nomogram. In addition, to evaluate the clinical applicability of the nomogram, decision curve analysis (DCA) was carried out. The total score of each patient was determined using the nomogram; the cut-off value by the ROC curve was delineated, the patients were divided into high-risk and low-risk groups, and the verification efficiency was evaluated.

### Continuous Variable Assignment

For continuous variables, the cut-off value was determined using a ROC curve, and the patients were divided into two groups according to the cut-off value (SPSS assigned values of 0 and 1). Cut-off values for the CEA level, age, and body mass index (BMI) were 2.43, 64.5, and 18.97, respectively.

## Results

### Baseline Characteristics

A total of 598 patients met the inclusion criteria; 426 and 172 patients were assigned to the training and verification cohorts, respectively (Figure 1). The clinicopathological characteristics of the patients are listed in Table 3. In the two cohorts, 290 (68.1%) and 109 (63.4%) cases were pathologically suggestive of locally advanced ESCC. There were no significant differences in age, sex, smoking, tumor location, BMI, and preoperative CEA level between the training and validation cohorts (*P *> 0.05). However, in patients with concomitant disease, lymph node enlargement, preoperative symptoms, and a gross aspect of the tumor, there were significant differences between the two groups (*P *< 0.05).

**Table 3:** Participant characteristics.

Variable	Total (*n* = 598)	Cohort, No. (%)		*P*-value
		Training (*n* = 426)	Validation (*n* = 172)	
Age (years) ±SD	59.17 ± 8.212	59.37 ± 8.384	58.67 ± 7.769	0.344
BMI (kg/m^2^) ±SD	22.18 ± 3.105	22.06 ± 3.188	22.45 ± 2.881	0.165
CEA (ng/mL) ±SD	2.86 ± 2.232	2.89 ± 2.311	2.78 ± 2.030	0.610
Sex, *n* (%)				0.680
Male	438 (73.2%)	116 (27.2%)	44 (25.6%)	
Female	160 (26.8%)	312 (72.8%)	128 (74.4%)	
Smoking, *n* (%)				0.110
Yes	324 (54.2%)	222 (52.1%)	102 (59.3%)	
No	274 (45.8%)	204 (47.9%)	70 (40.7%)	
Concomitant disease, *n* (%)				0.005
Yes	161 (26.9%)	101 (23.7%)	60 (34.9%)	
No	437 (73.1%)	325 (76.3%)	112 (65.1%)	
Tumor location, *n* (%)				0.396
Upper	52 (8.7%)	40 (9.4%)	12 (7.0%)	
Middle	294 (49.2%)	213 (50.0%)	82 (47.1%)	
Lower	252 (42.1%)	173 (40.6%)	79 (45.9%)	
Lymph node enlargement, *n* (%)				<0.001
Yes	284 (47.5%)	236 (55.4%)	48 (27.9%)	
No	314 (52.5%)	190 (44.6%)	124 (72.1%)	
Preoperative symptoms, *n* (%)				<0.001
Yes	516 (86.3%)	381 (89.4%)	135 (78.5%)	
No	82 (13.7%)	45 (10.6%)	37 (21.5%)	
Gross aspect				0.031
Early	194 (32.4%)	127 (29.8%)	67 (39.0%)	
Advanced	404 (67.6%)	299 (70.2%)	105 (61.0%)	
pTNM-stage, *n* (%)				0.269
Early	199 (33.3%)	136 (31.9%)	63 (36.6%)	
Advanced	399 (66.7%)	290 (68.1%)	109 (63.4%)	

*CEA, Carcinoembryonic antigen; BMI, body mass index.*

### Preoperative Independent Predictors

For the training cohort, univariate analysis showed that higher preoperative CEA level, presence of preoperative symptoms, lymph node enlargement, and advanced gross aspect indicated an advanced postoperative pathological stage (*P *< 0.1). On the other hand, multivariate analysis showed that higher preoperative CEA level (>2.43, OR: 2.093; 95% CI, 1.233–2.554; *P *= 0.006), presence of preoperative symptoms (OR: 2.737; 95% CI, 1.194–6.277; *P *= 0.017), lymph node enlargement (OR: 2.100; 95% CI, 1.243–3.550; *P *= 0.006), and advanced gross aspect (OR: 13.103; 95% CI, 7.689–23.330; *P *< 0.001) were independent predictors of occult locally advanced ESCC (Table 4).

**Table 4:** Logistic univariate and multivariate regression analysis of occult locally advanced ESCC based on preoperative data in the training cohort.

Variable	Univariable		Multivariable	
	OR (95% CI)	*P*-value	OR (95% CI)	*P*-value
Age		0.138		
≤64.5	reference			
>64.5	1.497 (0.878–2.552)			
BMI		0.657		
≤18.97	reference			
>18.97	1.149 (0.623–2.117)			
CEA		<0.001		0.006
≤2.43	reference		reference	
>2.43	2.401 (1.566–3.679)		2.093 (1.233–2.554)	
Sex		0.105		
Female	reference			
Male	1.448 (0.926–2.265)			
Smoking		0.102		
No	reference			
Yes	1.406 (0.935–2.117)			
Concomitant disease		0.853		
No	reference			
Yes	0.956 (0.593–1.540)			
Tumor location		0.580		
Upper	reference			
Middle	1.282 (0.628–2.616)	0.495		
Lower	1.040 (0.506–2.141)	0.914		
Lymph node enlargement		<0.001		0.006
No	reference		reference	
Yes	2.105 (1.392–3.183)		2.100 (1.243–3.550)	
Preoperative symptoms		<0.001		0.017
No	reference		reference	
Yes	5.189 (2.684–10.030)		2.737 (1.194–6.277)	
Gross aspect		<0.001		<0.001
Early	reference		reference	
Advanced	14.168 (8.659–23.510)		13.103 (7.689–23.330)	

*BMI, body mass index; OR, odds ratio; CI, confidence interval; CEA, Carcinoembryonic antigen.*

### Nomograms

**Figure 2:**
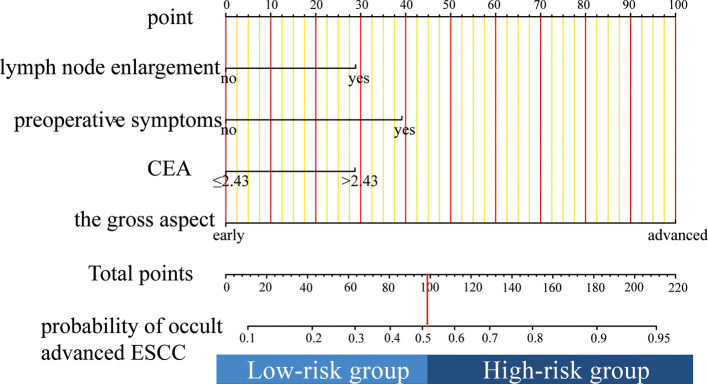
To use the nomogram, find the position of each variable on the corresponding axis, draw a line to the points axis for the number of points, add the points from all of the variables, and draw a line from the total points axis to determine the occult advanced ESCC probabilities at the lower line of the nomogram.

The nomogram was established based on the results of the multivariable analyses (Figure 2). The nomogram consisted of four variables and the corresponding score axis. The state of each variable corresponded to a score on the nomogram axis. The sum of the scores of all variables was the total score. This axis showed the risk of occult locally advanced ESCC on the prediction line below it.

### Development of a Web Server for Easy Access to Our New Model

An online version of our nomogram can be accessed at https://zhixinhuang.shinyapps.io/dynnomapp/. The predicted risks of occult locally advanced ESCC over time can be easily determined by inputting clinical features and reading output figures and tables generated by the web server.

### Calibration and Discrimination of the Nomogram

**Figure 3:**
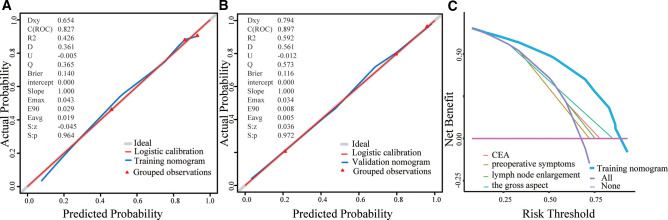
**A**, Validity of the predictive performance of the nomogram in estimating the risk of occult advanced ESCC presence in the training cohort (n = 426). **B**, Validity of the predictive performance of the nomogram in estimating the risk of occult advanced ESCC presence in the validation cohort (n=172). **C**, Decision curve for prediction of occult advanced ESCC. Pink line: assume no patient will have occult advanced ESCC; Purple line: assume all patients will have occult advanced ESCC; The x-axis and the y-axis were the threshold probability and the net benefit, respectively.

As shown in Figures 3A,B, the Brier scores of the training and validation cohorts were 0.140 and 0.116, respectively. Both calibration curves showed better acceptable consistency between the nomogram prediction and observation of locally advanced ESCC. The unadjusted C-indexes of the training and validation cohorts were 0.827 (95% CI, 0.782–0.872) and 0.897 (95% CI, 0.849–0.945), and the bootstrap-corrected C-indexes were 0.824 and 0.888, respectively.

### Clinical Utility of the Nomogram

DCA was performed to evaluate the clinical utility of the nomogram based on the net benefits at different threshold probabilities. Compared with the preoperative CEA level, presence of preoperative symptoms, lymph node enlargement, and advanced gross aspect, the increased net benefit of the nomogram was the largest, which indicated that the nomogram was a reliable clinical tool for predicting occult locally advanced ESCC (Figure 3C).

### Risk of Occult Locally Advanced ESCC Based on the Nomogram Scores

We calculated a cut-off value of 98 based on the ROC and divided the patients into two groups: high-risk and low-risk groups. The sensitivity, specificity, positive predictive value, and negative predictive value for distinguishing the presence of occult locally advanced ESCC were 87.6%, 66.9%, 84.9%, and 71.7% in the training cohort, and 87.2%, 81.0%, 88.8%, and 78.5% in the validation cohort, respectively (Table 5).

**Table 5:** Accuracy of the prediction score of the nomogram for estimating the risk of occult locally advanced ESCC.

Variable	Value	
	Training cohort	Validation cohort
C-index, AUC	0.827 (95% CI, 0.782–0.872)	0.897 (95% CI, 0.849–0.945)
Bootstrap C-index	0.824	0.888
Cutoff score	98	98
Sensitivity, %	87.6%	87.2%
Specificity, %	66.9%	81.0%
Positive predictive value, %	84.9%	88.8%
Negative predictive value, %	71.7%	78.5%
Positive likelihood ratio	2.65	4.58
Negative likelihood ratio	0.19	0.16

*AUC, Area under ROC curve.*

### Applicability of the Prediction Model to T1/T2 ESCC

In clinical practice, neoadjuvant therapy is recommended once there is lymph node metastasis for patients with T1/T2. Therefore, this study explored the applicability of this prediction model to these patients. In the training cohort, there were 187 (43.90%) patients with pT1 or pT2; among them, there were 56 patients with lymph node metastasis. In the validation cohort, there were 88 (51.16%) patients with pT1 or pT2, 25 of whom had lymph node metastasis. The sensitivity, specificity, positive predictive value, and negative predictive value for distinguishing the presence of occult locally advanced ESCC were 65.5%, 65.1%, 44.4%, and 81.5% for the training cohort and 64.0%, 79.7%, 57.1%, and 83.9% for the validation cohort, respectively (Table 6).

**Table 6:** The accuracy of nomogram in T1/T2 staging of ESCC.

		pT1/ pT2 and pN+	pT1/ pT2 and pN0	Total
High-risk group (training cohort)		36	45	81
Low-risk group (training cohort)		19	84	103
Total (training cohort)		55	129	184
High-risk group (validation cohort)		16	12	28
Low-risk group (validation cohort)		9	47	56
Total (validation cohort)		25	59	84
Sensitivity, %	training cohort	65.5%		
	validation cohort	64.0%		
Specificity, %	training cohort	65.1%		
	validation cohort	79.7%		
PPV, %	training cohort	44.4%		
	validation cohort	57.1%		
NPV, %	training cohort	81.6%		
	validation cohort	83.9%		
PLR	training cohort	1.88		
	validation cohort	3.14		
NLR	training cohort	0.53		
	validation cohort	0.45		

*PPV, Positive predictive value; NPV, Negative predictive value; PLR, Positive likelihood ratio; NLR, Negative likelihood ratio.*

### Applicability of the Prediction Model to T3/T4 ESCC

In the training cohort, 235 patients had pT3 or pT4, while in the validation cohort, 84 patients had pT3 or pT4. The nomogram matched the final surgical pathology in 218/235 patients for concordance rates of 92.8% and 94.0% in the training and validation cohorts, respectively.

## Discussion

For locally advanced ESCC, neoadjuvant treatment is more reasonable than surgical treatment. Unfortunately, current preoperative examinations cannot accurately identify locally advanced ESCC. The focus of this study was to conduct an assessment for patients with negative lymph nodes before surgery to minimize the occurrence of occult locally advanced ESCC. We collected independent predictors of occult locally advanced ESCC and used them to construct a predictive model to quantify the risk of occult locally advanced ESCC.

### Independent Predictors of Occult Locally Advanced ESCC

Multivariate analysis showed that a higher preoperative CEA level, obvious preoperative symptoms, lymph node enlargement, and an advanced gross aspect are independent predictors of occult locally advanced ESCC. CEA is a glycoprotein involved in cell adhesion that is usually produced in gastrointestinal tissues during fetal development (15). Studies have shown that changes in CEA levels reflect the tumor burden and are affected by several factors (16). Marco et al. showed that CEA levels were significantly higher in patients with unresectable advanced EC and verified that preoperative CEA levels were independent predictors of occult locally advanced EC (9), similar to the findings of the current study. Due to the low expression of CEA in ESCC, the common values of CEA are of little significance. Our research showed that a preoperative CEA level >2.43 is indicative of occult locally advanced ESCC. Additionally, obvious preoperative symptoms may indicate the progress of EC. In our study, the risk of locally advanced ESCC in patients with preoperative symptoms was 2.7 times that of patients without symptoms (OR: 2.737; *P *= 0.006), similar to the findings of Sara et al. (17). Lymph node enlargement on ultrasound or CT images in patients with ESCC is usually considered to identify abnormal lymph node size that is not associated with signs of lymph node metastasis; however, postoperative pathology often confirms lymph node metastasis. Lymph node enlargement can be caused by infection, cancers, reactive hyperplasia, and abnormal cell proliferation and metabolism (18, 19). In patients with ESCC, tumors can metastasize to the entire body through lymph nodes and lymph vessels. Therefore, lymph node enlargement in the drainage area of ESCC mainly suggests lymph node reactive hyperplasia or tumor metastasis. There may be micro-metastases in the enlarged lymph nodes, when the cancer cells have reached the corresponding lymph nodes but have not yet formed obvious metastases, which is difficult to find on preoperative examination (20, 21). Our research also showed that among patients with negative lymph node metastasis, those with enlarged lymph nodes had a higher tumor pT-stage (*P *= 0.003). This may be because the inflammatory microenvironment in the lymph nodes plays a role in promoting tumors. The stimulation of tumor-associated antigens and secreted cytokines causes various inflammatory cells to gather in the lymph nodes of the drainage area (22). The gross aspect of the tumor under endoscopy was often regarded as the most intuitive method for judging the depth of tumor invasion. When the gross aspect is superficial, the probability of lymph node metastasis is considered low. Our research further affirmed the positive role of endoscopy in judging the general type of tumor, which is consistent with the findings of previous studies. However, we believe that combining other factors to judge preoperative occult locally advanced EC will be more accurate and reliable.

### Applicability of the Prediction Model to T1/T2 ESCC

For patients with shallow tumor invasion but lymph node metastasis (cT1/cT2 and any N+), neoadjuvant therapy is recommended first. Therefore, our study also explored the applicability of this model to these patients. After dividing the risk group, the sensitivity and specificity for distinguishing the presence of occult locally advanced esophageal cancer were 65.5% and 65.1% for the training cohort and 64.0% and 79.7% for the validation cohort, respectively. The research of David Bunting et al. showed that the sensitivity of EUS for judging lymph node metastasis is only 34.5%, while the specificity is 88.2% (23). Similarly, De Nucci's study showed that the diagnostic sensitivity of preoperative EUS in patients with postoperative pathological confirmation of T2N0 was only 65.2% (24). The prediction performance of our model is higher than that of EUS. Our model is aimed at screening patients with negative lymph nodes before surgery, and the focus is on reducing the missed diagnosis rate. For patients in the high-risk group, we recommend further examinations such as PET-CT and EUS-guided fine-needle aspiration before surgery (25, 26).

### Applicability of the Prediction Model to T3/T4 ESCC

For patients with a preoperative stage of T3/T4, neoadjuvant therapy can facilitate subsequent radical resection. The nomogram results matched the final surgical pathology results with a concordance rate of 92.8% for the training cohort and a concordance rate of 94.0% for the validation cohort. In Yang's study, the agreement rate of EUS in accurately identifying T3/T4 patients was only 68.6% (27). In Shi's study, the agreement rate of EUS in accurately identifying T3/T4 patients was only 88.7% (28). Our model showed a higher discrimination performance than EUS.

### Predictive Model Establishment and Verification

The nomogram model is widely used in the medical field as it can integrate and quantify multiple clinical and pathological indicators and can be used to visualize the research results. In this study, a nomogram for predicting preoperative occult ESCC was constructed based on multivariate logistic regression analysis. With self-sampling internal verification, the C-index/AUC showed that this nomogram had good predictive performance; for the calibration curve, the high degree of fit suggested that the prediction model had a higher calibration. We combined the ROC curve cut-off values to divide the patients into high- and low-risk groups. Both the training and validation cohorts showed good sensitivity and specificity, which proved that the predictive model had good clinical applicability.

This study had some limitations. To the best of our knowledge, this is the first study to establish a predictive model for preoperative occult locally advanced ESCC; however, because the design research cycle is long and there is no data verification from other centers, more direct evidence still needs to be confirmed by prospective research. Furthermore, to improve the predictive ability of the predictive model, we only examined patients with postoperative pathological confirmation of ESCC, which limited the scope of application of this prediction model. Nonetheless, the number of patients analyzed was relatively large, which can guarantee the reliability of the model.

In conclusion, we confirmed that higher preoperative CEA levels, presence of preoperative symptoms, lymph node enlargement, and locally advanced gross aspect are independent predictors of occult locally advanced ESCC. We constructed a nomogram to predict the risk of occult locally advanced ESCC in patients with negative lymph nodes during preoperative examinations; the tool can be supplemented with preoperative examination to improve the reliability of preoperative staging.

## Data Availability

The raw data supporting the conclusions of this article will be made available by the authors, without undue reservation.
